# The Role of Neuropilin-1 (NRP-1) in SARS-CoV-2 Infection: Review

**DOI:** 10.3390/jcm10132772

**Published:** 2021-06-24

**Authors:** Monika Gudowska-Sawczuk, Barbara Mroczko

**Affiliations:** 1 Department of Biochemical Diagnostics, Medical University of Bialystok, Waszyngtona 15A St., 15-269 Bialystok, Poland; mroczko@umb.edu.pl; 2 Department of Neurodegeneration Diagnostics, Medical University of Bialystok, Waszyngtona 15A St., 15-269 Bialystok, Poland

**Keywords:** NRP-1, SARS-CoV-2, coronavirus, COVID-19, host cell

## Abstract

Severe acute respiratory syndrome coronavirus 2 (SARS-CoV-2), discovered in 2019, is responsible for the global coronavirus disease 19 (COVID-19) pandemic. The main protein that interacts with the host cell receptor is the Spike-1 (S1) subunit of the coronavirus. This subunit binds with receptors present on the host cell membrane. It has been identified from several studies that neuropilin-1 (NRP-1) is one of the co-receptors for SARS-CoV-2 entry. Therefore, in this review, we focus on the significance of NRP-1 in SARS-CoV-2 infection. MEDLINE/PubMed database was used for a search of available literature. In the current review, we report that NRP-1 plays many important functions, including angiogenesis, neuronal development, and the regulation of immune responses. Additionally, the presence of this glycoprotein on the host cell membrane significantly augments the infection and spread of SARS-CoV-2. Literature data suggest that NRP-1 facilitates entry of the virus into the central nervous system through the olfactory epithelium of the nasal cavity. Moreover, published findings show that interfering with VEGF-A/NRP-1 using NRP-1 inhibitors may produce an analgesic effect. The review describes an association between NRP-1, SARS-CoV-2 and, inter alia, pathological changes in the retina. Based on the published findings, we suggest that NRP-1 is a very important mediator implicated in, inter alia, neurological manifestations of SARS-CoV-2 infection. Additionally, it appears that the use of NRP-1 inhibitors is a promising therapeutic strategy for the treatment of SARS-CoV-2 infection.

## 1. Introduction

Human coronaviruses were first discovered in the 1960s and the *Coronaviridae* family of order Nidovirales was established in the Taxonomy of Viruses in 1975 [1,2]. In February 2003, a coronavirus (SARS-CoV) was identified as the etiological factor of severe acute respiratory syndrome (SARS) with a case fatality rate of approximately 10% [3]. Tragically, in November 2019, a pandemic of severe acute respiratory syndrome coronavirus 2 (SARS-CoV-2) broke out. The first patient with a probable case of SARS-CoV-2 was admitted to hospital in Wuhan City, China. In 2020, the WHO announced a new, official name of the acute infectious disease caused by the abovementioned virus—coronavirus disease (COVID-19). By the end of April 2021, there had been around 150,259,386 confirmed cases of the disease, of which 3,182,057 had resulted in patient death [4,5].

SARS-CoV-2 spreads rapidly through person-to-person contact, when respiratory droplets from an individual infected with the virus enter the respiratory system of someone who is not infected [6]. The structure of coronaviruses is very characteristic due to the presence of crown-like spikes on their surface. Analysis of the coronavirus genome has revealed that the sequence of SARS-CoV-2 is 79.5–82.0% identical to SARS-CoV [7,8]. SARS-CoV-2 has very large, single-stranded, 26–32 kb RNA [8,9]. The sequence of nucleotides in RNA determines the sequence of four structural proteins—spike (S), nucleocapsid (N), membrane (M) and envelope (E)—which are very important components of this betacoronavirus [9,10].

In recent months, extensive experimental research has been conducted around the world on a drug to treat COVID-19 and vaccines against the SARS-CoV-2 coronavirus. However, vaccine effectiveness depends on many factors, inter alia, molecular mechanisms of viral entry into the host cell.

Viruses that cause infection directly interact with the immune system. SARS-CoV-2 attach to the factor located on a host cell and the response outcome is highly dependent on cell type. Interaction of SARS-CoV-2 surface proteins with specific receptors initializes endocytosis. Coronavirus entry into host cells is associated with interferon (IFN) production. Moreover, it has been reported that humoral immune responses are similar to other virus infections, and are associated with specific IgM and IgG and synthesis. At the beginning of the infection, B cells elicit a response against the nucleocapsid protein, whereas antibodies against the spike protein are produced after about one week from the appearance of symptoms [11,12,13].

Interaction of SARS-CoV-2 surface proteins with specific receptors initializes endocytosis. Virus entry into host cells is initiated by attachment of the spike protein to specific receptors found in, inter alia, the lungs, kidneys, heart or brain. Typically, the receptor-binding domain (RBD), which is found on the surface of SARS-CoV-2, binds to human angiotensin-converting enzyme 2 (ACE2), whereas transmembrane serine protease 2 (TMPRSS2) is a protease important for S proteolytic processing and priming during infection. Additionally, it has been indicated that neuropilins are co-receptors that promote the entry of SARS-CoV-2 into the cell [14,15,16]. The Neuropilin family consists of two members—neuropilin-1 (NRP-1) and neuropilin-2 (NRP-2)—which exert an important impact on lymphangiogenesis, angiogenesis or axon guidance [17]. However, there have been no studies investigating the role of NRP-2 in SARS-CoV-2 infection in the available literature. Therefore, in the present review, we summarize and refer to a number of original papers exploring the significance of NRP-1 as a cell surface entry mediator for SARS-CoV-2.

## 2. Methods

### Literature Search and Data Extraction

We performed a comprehensive literature search covering the period up to 23 April 2021 using the MEDLINE/PubMed electronic database with the following search strategy: key words “SARS-CoV-2” (76,159 studies). When we used the key words “SARS-CoV-2 AND receptor”, a total of 5216 papers were found. A search including the key words “SARS-CoV-2 AND neurpilin-1” produced a total of 31 papers. The next step involved limiting the search to human studies written in English and the exclusion of review papers and duplicates. Thus, 17 original publications and letters to editor were included in the study. All studies found were published between August 2020 and April 2021.

## 3. Results

**Figure 1:**
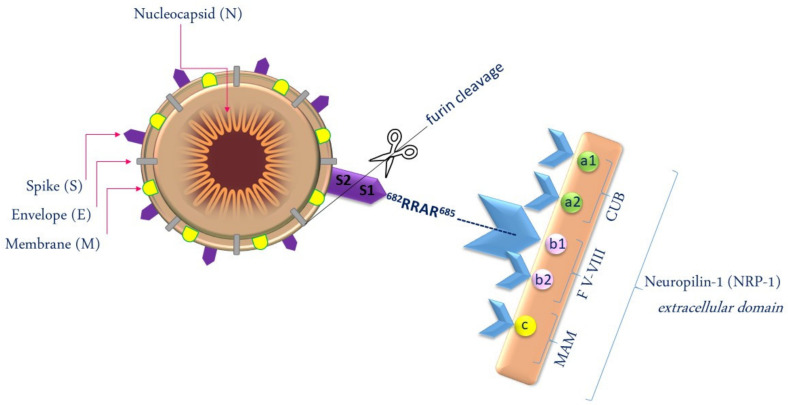
SARS-CoV-2 entry into host cells depends on NRP-1.

NRP-1 plays a diverse range of functions in cell proliferation, immunity, and physiological and pathological angiogenesis. It has also been indicated that NRP-1 plays a fundamental role in axon and neuronal development [18], and that it may be involved in the pathogenesis of SARS-CoV-2. The presence of NRP-1 facilitates the process of SARS-CoV-2 entry into the host cell. SARS-CoV-2 entry into host cells is initiated, inter alia, by attachment to NRP-1 and is followed by conformational changes of the viral spike protein. Proteolytic cleavage with the host furin protease is principally the process of breaking the spike protein into two polypeptides: Spike-1 (S1) and Spike-2 (S2). The S1 subunit has a specific amino acid sequence of the C-terminal peptide: Arg-Arg-Ala-Arg (^682^RRAR^685^) and the S1 subunit, which conforms to the C-end-rule (CendR), and binds to a specific NRP-1 or NRP-2 molecule on the target cell. It has been described that the extracellular regions of neuropilins are composed of an MAM domain (the domain consists of meprin, A-5 protein and receptor protein–tyrosine phosphatase mu), two complement-like binding (CUB) domains (a1 and a2) and two coagulation factor domains (b1 and b2). Among the domains listed above, coagulation factor domain b1 is responsible for binding C-end-rule peptides (Figure 1) [19,20,21].

Daly et al., using X-ray crystallography, demonstrated that NRP-1 precipitated Spike-1 protein [19]. Interestingly, it was shown that NRP-1 binds more strongly than the S1 subunit to the host cell surface. Therefore, it was suggested that NRP-1 leads to destabilization of the S protein complex and activates the exit of S2 from the S1 subunit. Additionally, S2 is released from S1 at the initial step of NRP-1 binding. On the other hand, it has been shown that the S1 subunit is responsible for receptor binding, whereas the S2 domain is responsible for fusion and replication, and that it is the most conserved region of the Spike protein [19,22].

It has been demonstrated that deletion of the CendR motif reduces the association between S1 and NRP-1. Additionally, neuropilin-1 depletion results in halving the SARS-CoV-2 uptake in comparison to control cells. This proves that NRP-1 facilitates SARS-CoV-2 entry into host cells. Hence, cells and tissues enriched for NRP1 receptors exhibit enhanced viral infectivity risk [19,23,24]. Furthermore, some authors have suggested that NRP-1 is a cofactor of ACE2 and S protein binding, but without the presence of ACE2, SARS-CoV-2 has very low infectivity. It should also be pointed out that SARS-CoV-2 does not infect cells that are NRP1-positive but ACE2-negative. On the contrary, SARS-CoV-2 infects ACE2-positive cells in which NRP1 is knocked out [23,24]. Cantuti-Castelvetri et al. generated lentiviral particles pseudotyped with S protein found in SARS-CoV-2. They used embryonic kidney 293T cells (HEK-293T) which are naturally devoid of ACE2 and NRP-1 receptors. Next, using plasmids, both transcripts were transferred into HEK-293T cells. The authors observed that NRP-1 only with the coexpression of ACE-2 and/or TMPRSS2 enhanced infection of coronavirus, as opposed to NRP-1 alone. Therefore, they suggested that NRP-1 is an entry cofactor of ACE2 which facilitates communication between SARS-CoV-2 and ACE2 [25]. A study by Alnomasy investigated the interaction between the receptor-binding domain of the coronavirus and the b1 domain of NRP-1. The author performed in silico analysis using the Protein Data Bank to download RBC and b1 structures. It was observed that the spike RBD domain of SARS-CoV-2 and the b1 domain of NRP-1 interact via amino acids: Gln^280,^ Asp^289^, Tyr^322^, Arg^323^, Trp^325^, Gln^327^, Asp^329^, Lys^359^ and Asp^361^. However, it ought to be emphasized that computational modelling is predictive and should always be confirmed by experimental analysis [20].

It is well known that in patients with respiratory infections of SARS-CoV-2, the disease may be associated with neurological manifestations, including headaches, dizziness, hallucinations or motor coordination disturbances [26]. A study by Mao et al. revealed that 45.5% of patients with confirmed COVID-19 infection showed signs and symptoms of central nervous system (CNS) disorders [27]. It has been also observed that SARS-CoV-2 infection correlates with increased coagulation. Knowing that coagulation is also modulated by NRP-1, we carefully suggest that a key component of the pathology in CNS may be associated with an imbalanced clotting response in patients with COVID-19.

It has been suggested that SARS-CoV-2 enters the CNS through the olfactory epithelium of the nasal cavity. Therefore, in order to examine the presence of NRP-1 in specific cells of the human brain, Davies et al. measured its RNA expression through single-cell RNA sequencing. They observed the expression of NRP-1 in endothelial cells, macrophages, neurons, fetal astrocytes, oligodendrocytes, although it was highest in mature astrocytes. Using second RNA sequencing, they examined brain regions with NRP-1 expression. RNA expression of NRP-1 was highest in the hippocampal formation in comparison to the olfactory region, amygdala, basal ganglia, cerebral cortex, thalamus, hypothalamus, midbrain, cerebellum, retina, pons and medulla [28]. Similarly, other authors used antibodies against the spike protein to demonstrate a markedly elevated expression of NRP-1 in infected olfactory epithelial cells from human COVID-19 autopsies. There is evidence that NRP-1 enhances the entry of SARS-CoV-2 into the CNS [25].

Interestingly, Heinonen et al. determined the expression of SARS-CoV-2 entry receptors in newborns and observed that NRP-1 expression was decreased in newborns in comparison to adults. They also observed that the expression of other SARS-CoV-2 co-receptors, ACE2, TMPRSS2 and neuropilin-2 (NRP-2), were lower in the nasal epithelium of newborns. This may explain why children generally have none or mild symptoms of the SARS CoV-2 infection [29].

It is well known that regulatory T (T reg) cells control the immune response to an antigen. It has been demonstrated that NRP-1 is found on natural regulatory T cells which can be divided into thymic and peripheral. Peripheral T reg cell synthesis occurs mainly in response to the transcription factor Foxp3 stimulated by antigens, whereas thymus studies have showed that on the surface of mature Foxp3+ T regulatory cells, expression of NRP-1 is the highest. T reg cells can also be expressed on cancer cells or endothelial cells. T cells are responsible for suppressing too severe an autoreactive immune response, and hence the use of these cells in the treatment of many diseases including colorectal or breast cancer is presently undergoing clinical trials. It has been described that the therapeutic manipulation of T lymphocytes is dependent on surface receptors including NRP-1 [30,31].

Due to the fact that NRP-1 is involved in endothelial-dependent immune responses in the human brain, Mone et al. attempted to identify microRNAs (miRNAs) that specifically target NRP-1 in brain microvascular endothelial cells representing the blood–brain barrier. Using Targetscan 7.2, the authors identified hsa-miR-24–3p (miR-24), which is probably a modulator of neuropilin-1 mRNA expression [32]. Therefore, miR-24 has been indicated as a potentially therapeutic target focused on the reduction in endothelium-dependent inflammation. Additionally, it has been found that miR-24 decreases the blood–brain barrier permeability in response to vascular endothelial growth factor (VEGF), which has been indicated as one of the most studied ligands of NRP-1 [32]. Moreover, it has been revealed that VEGF-A sensitizes nociceptor activity which initiates the sensation of pain in the central nervous system [33,34]. Considering the above and that VEGF-A levels are elevated in, inter alia, bronchial fluid from patients infected with SARS-CoV-2, Moutal et al. attempted to establish whether the S protein of SARS-CoV-2 could block VEGF-A/NRP-1 signaling. Interestingly, they observed that interfering with VEGF-A/NRP-1 using the S protein or EG00229 (inhibitor of NRP-1) leads to the suppression of spinal synaptic activity and a decrease in electrogenic currents in afferent neurons [34]. NRP-1 has been studied as a modulator of VEGF-mediated angiogenesis. It has been demonstrated by Parker et al. that VEGF-A specific binding may depend on NRP-1 residues in the b1 domain. Hence, it has been indicated that the use of soluble neuropilin has utility as a broad-spectrum NRP ligand inhibitor [35]. It has been suggested that VEGF-A/NRP-1 inhibition in pulmonary vagal neurons by the SARS-CoV-2 S protein may have an analgesic effect [34]. Moreover, some authors indicate that subversion of VEGF-A/NRP-1 may be associated with an asymptomatic course of the disease and should be considered a potential treatment of neuropathic pain as well as the potential of inhibiting SARS-CoV-2 virus entry [34,36]. It may be responsible for olfactory dysfunctions such as anosmia observed in patients with SARS-CoV-2 infection. In addition, Hopkins et al. hypothesize that there is a link between survival and olfactory dysfunction, because the majority of patients recover their loss of smell. However, further studies are necessary to elucidate the association between NRP-1 expression and the severity of olfactory disorders [37].

Comparable results were obtained by Perez-Miller et al., who revealed that interference of the VEGF-A/NRP-1 pathway by the SARS-CoV-2 S protein interferes with pain signaling. The authors identified nine small molecules with drug-like properties that occupy the CendR binding site on the b1 domain on the NRP-1 surface and induce the phosphorylation of VEGFR2. Furthermore, they demonstrated that six of them inhibit VEGF-A/NRP-1 binding more effectively than EG00229, and two of them inhibit the entry and fusion into the host cell of the SARS-CoV-2 S protein [36,38]. It has also been described that the main amino acids in the interaction between NRP-1 and EG00229 are: Thr316, Asp320, Ser346, Thr349 and Tyr353 [39]. Therefore, spinning forward, Vique-Sánchez et al. performed molecular docking to determine whether other compounds are able to inhibit the interaction of NRP-1 with the S1 protein of SARS-CoV-2. Using the crystallographic structure of NRP-1, they indicated 10 molecules (N1–N10) as potential binding inhibitors of the b1 region with the S1 region. Additionally, these compounds cause greater inhibition in comparison to the most examined NRP-1 inhibitor-EG00229. The authors suggest that the molecules identified in their study are more specific, and hence have higher binding value. It is equally important that N1–N10 are probably safe for use in humans, which was by validated by toxicity prediction web servers [40]. However, this is only a computational prediction, and it should be confirmed by future experimental evidence. The discovery of new NRP-1 inhibitors may be of great significance in the treatment of SARS-CoV-2 infection in the future.

It has been reported that NRP-1 and NRP-2 are also critical host factors for cytomegalovirus (CMV) infection in epithelial or endothelial cells. It has been observed that the proportion of individuals infected with CMV increases with age, similarly to the mortality rate of SARS-CoV-2 which also increases with age, and that CMV infection is linked to immune dysfunction, inter alia, the chronic activation of T cells. Therefore, it has been suggested that CMV infection is a negative risk factor for patient’s clinical status following SARS-CoV-2 infection [41,42,43,44].

Moreover, it has been observed that excessive oxidative stress may be an important factor in the development of obesity. Interestingly, in endothelial cells, NRP-1 promotes mitochondrial function by preventing iron accumulation and oxidative stress. On the other hand, NRP-1 loss leads to iron accumulation, the inhibition of cellular growth, and immunosenescence. Issitt T et al. observed that treatment with the mitochondria-targeted antioxidant of NRP-1–deficient endothelial cells inhibits superoxide production and protects from cellular senescence [45,46]. It is well established that heart diseases, hypertension, or diabetes are high-risk conditions for severe COVID-19 disease. Therefore, taking into consideration the fact that polycystic ovary syndrome (PCOS) is very commonly associated with the metabolic syndrome, obesity and insulin resistance, and that enhanced activity of the renin–angiotensin system (RAS) is observed in the metabolic syndrome, Moin ASM et al. suggest that PCOS may correlate with the circulating soluble NRP-1 (sNRP-1) level [24]. The authors examined the levels of NRP1, RAS-related proteins (ACE2, Renin, Angiotensinogen (AGT)) and VEGF in 146 PCOS patients and almost 100 controls using the Slow Off-rate Modified Aptamer-scan plasma protein measurement. They observed that circulating NRP-1 was decreased in PCOS, whereas VEGF levels did not differ between PCOS and healthy women. Moreover, sNRP-1 was negatively correlated with angiotensinogen, but not with VEGF, ACE2 or renin. Therefore, the authors carefully concluded that lower sNRP-1 levels with unchanged VEGF may reflect in higher endogenous membrane-bound NRP-1 in women with PCOS. Consequently, it may result in increased SARS-CoV-2 infectivity. Moreover, the negative correlation between sNRP-1 and angiotensinogen may indicate that enhanced RAS activation increases the risk of COVID-19 [24].

Furthermore, SARS-CoV-2 infection may lead to ophthalmological changes. Hence, it has been suggested that NRP-1 may be responsible for retinal diseases and conditions. SARS-CoV-2 infection may cause hyperreflective lesions in retinal ganglion cells of the eye [47]. Thus, El-Arabey et al. analyzed a single cell of retinal bipolar drop-seq [47,48]. Examination of NRP-1 expression revealed that this host factor is present in amacrine cells, retinal bipolar neurons and Müller glia cells. Considering that Müller glia cells facilitate hyperreflective lesions in the retina in accordance with VEGF-A, the authors investigated the potential role of NRP-1 in this pathological process. Their analysis revealed that SARS-CoV-2 in association with NRP-1 triggers microhemorrhages and hyperreflective lesions in the retina. Therefore, the authors suppose that NRP-1 is a mediator for several retinal findings in patients with COVID-19 [48].

Interestingly, a study by Zhao et al. demonstrated that chicken ovalbumin upstream promoter-transcription factor 2 (COUP-TF2) increases NRP-1 expression, which contributes to lung vascular repair after viral pneumonia. The study revealed that ligand-dependent NRP-1 is associated with angiogenesis and the migration of endothelial cells, and is directly targeted by COUP-TF2. Therefore, it was indicated that therapy of pulmonary vascular endothelium damage caused, inter alia, by SARS-CoV-2, can be based on the stabilization of COUP-TF2, which has an impact on NRP-1 [49].

## 4. Conclusions

In the present review, we analyzed data from manuscripts published between August 2020 and April 2021. Based on the published findings, we observed that NRP-1 is a very important co-receptor for SARS-CoV-2 entry into the host cell. It was also observed that the presence of NRP-1 on the host cell surface facilitates the spread of SARS-CoV-2 infection and may enable entry of the virus into, inter alia, the brain. Moreover, it appears that the use of NRP-1 or VEGF-A/NRP-1 inhibitors should be considered potential analgesic and antiviral therapy. Finally, our review demonstrates that the significance of NRP-1 in SARS-CoV-2 should be investigated further to confirm its role in coronavirus infection.
